# Supplementary material for: Exploring the association between precipitation and population cases of ocular toxoplasmosis in Colombia
Source: PLoS Negl Trop Dis. 2022 Oct 5;16(10):e0010742. doi: 10.1371/journal.pntd.0010742 (PMC9534415; doi:10.1371/journal.pntd.0010742)
Supplement: S1 Fig — (DOCX) [file pntd.0010742.s001.docx]

**S1 Figure:**  Cumulative-response curves for the association between precipitation and new cases of Ocular Toxoplasmosis.

| **Choco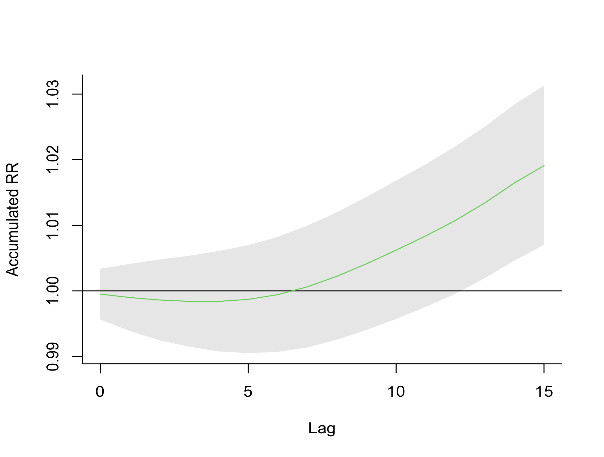** | **Cundinamarca**  **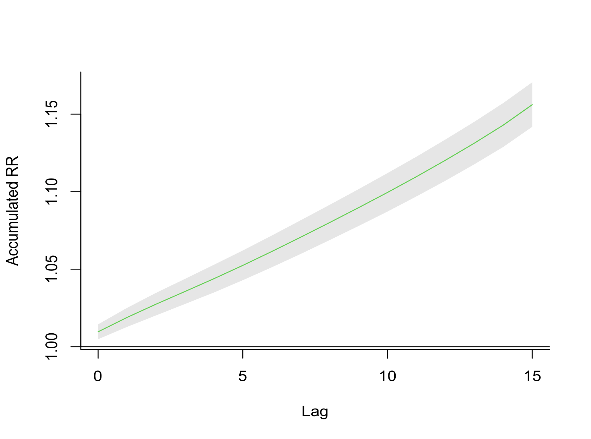** |
| --- | --- |
| **Antioquia 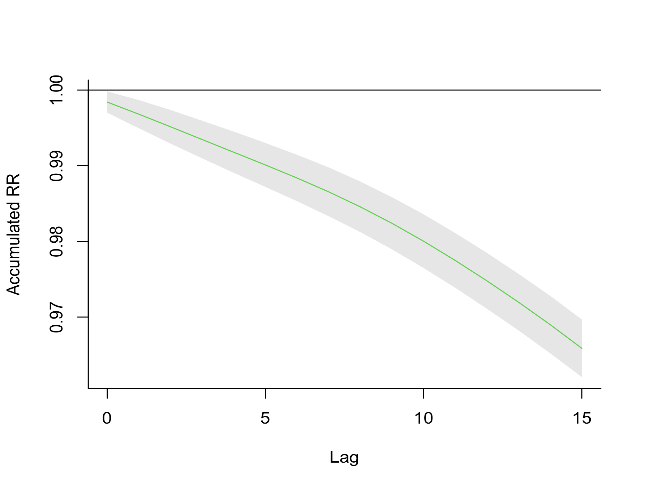** | **Córdoba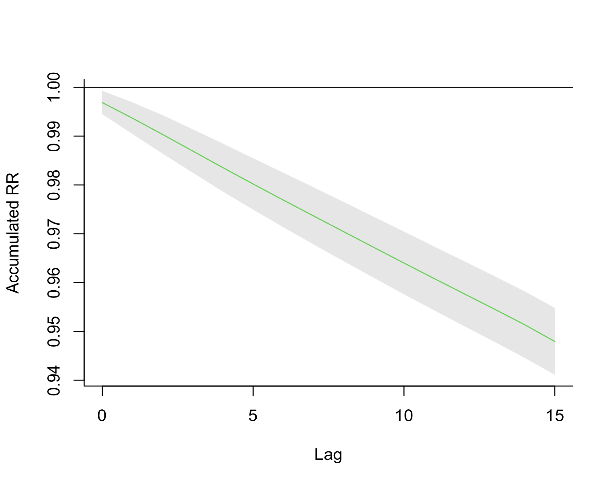** |
| **Guajira**  **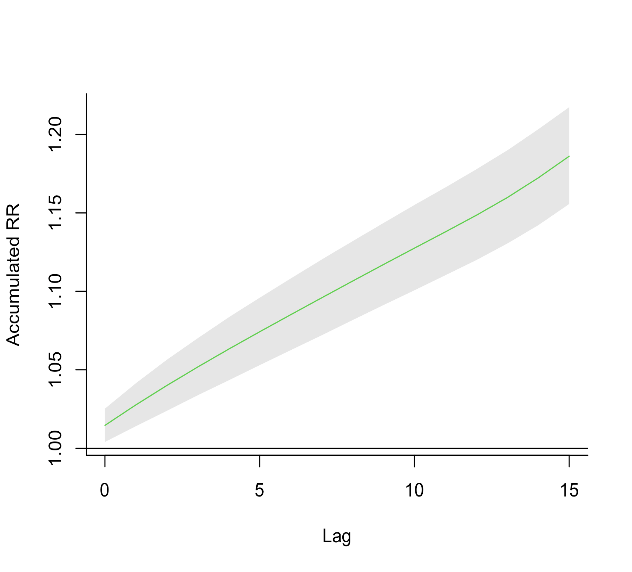** | **Tolima**  **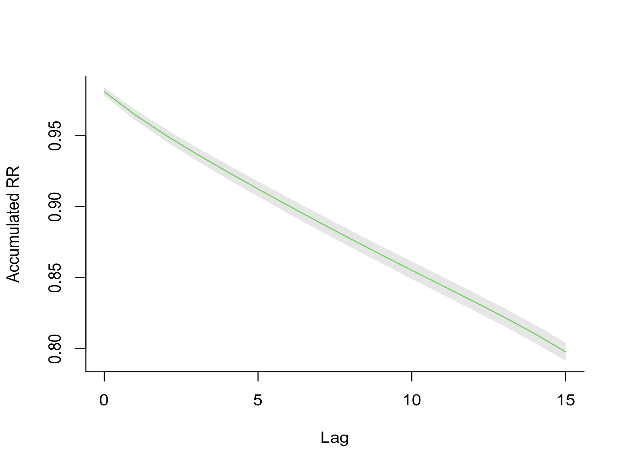** |
| **Santander**  **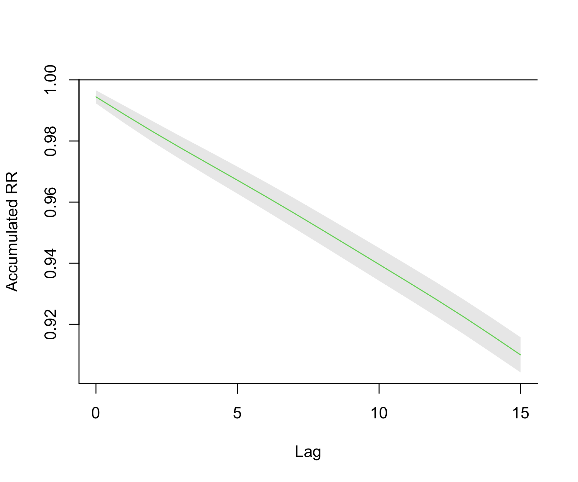** | **Sucre**  **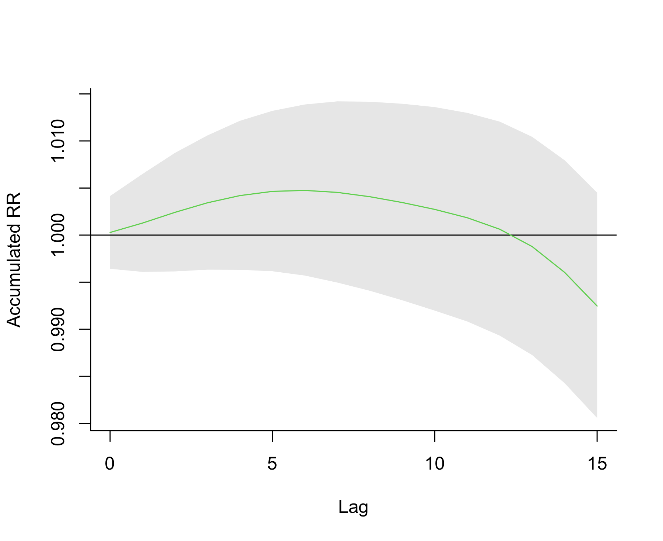** |
| **Cesar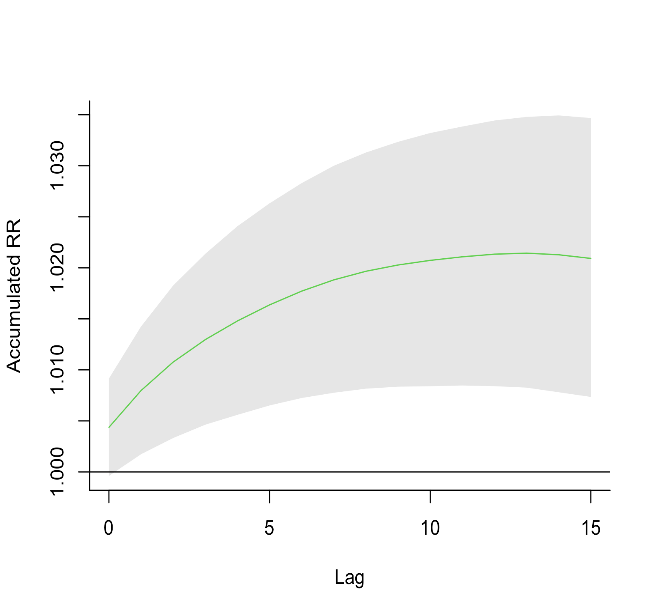** | **Atlántico**  **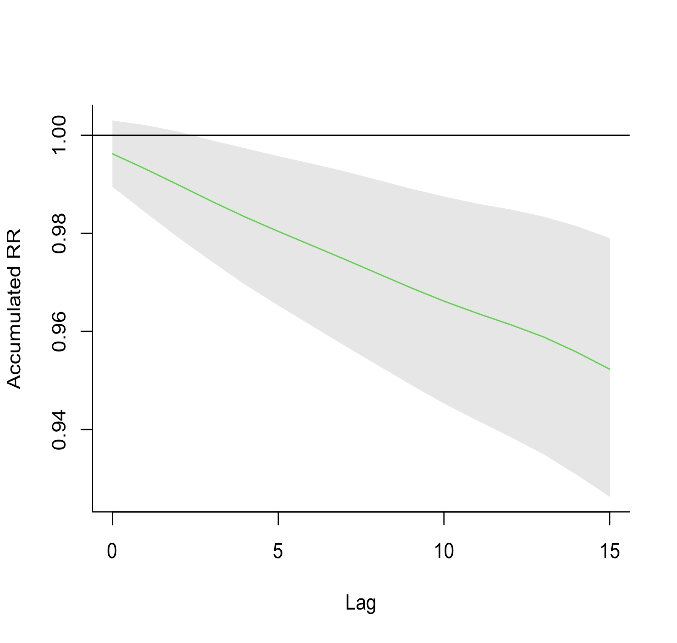** |
| **Huila**  **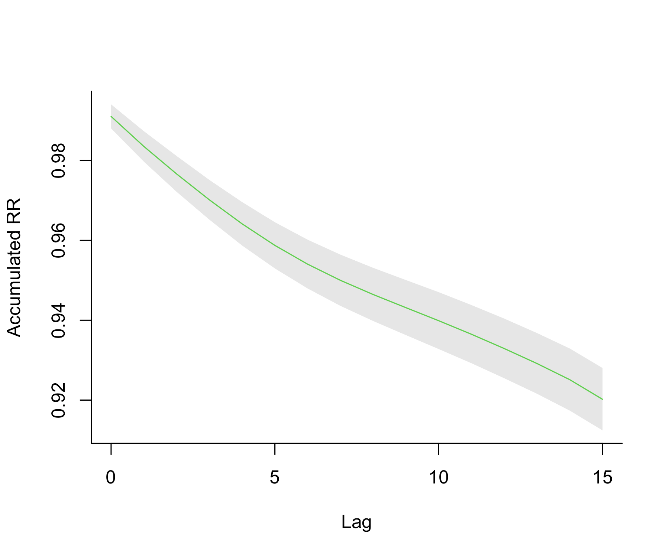** | **Bolívar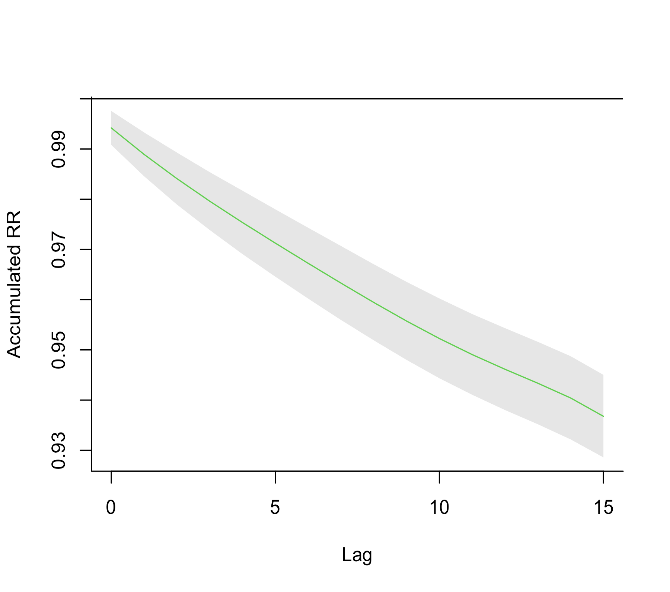** |
| **San Andrés 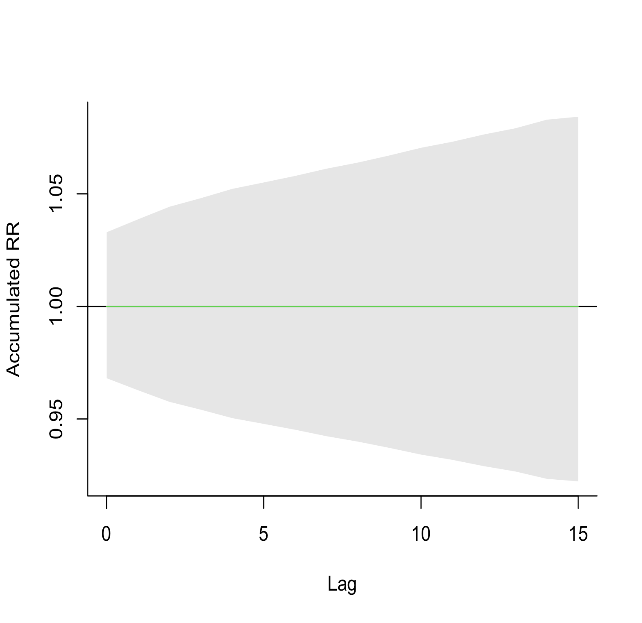** | **Magdalena**  **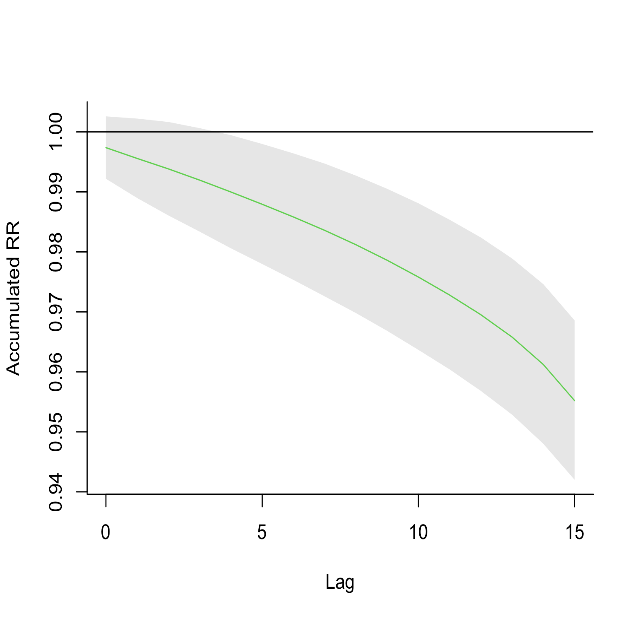** |
| **Cauca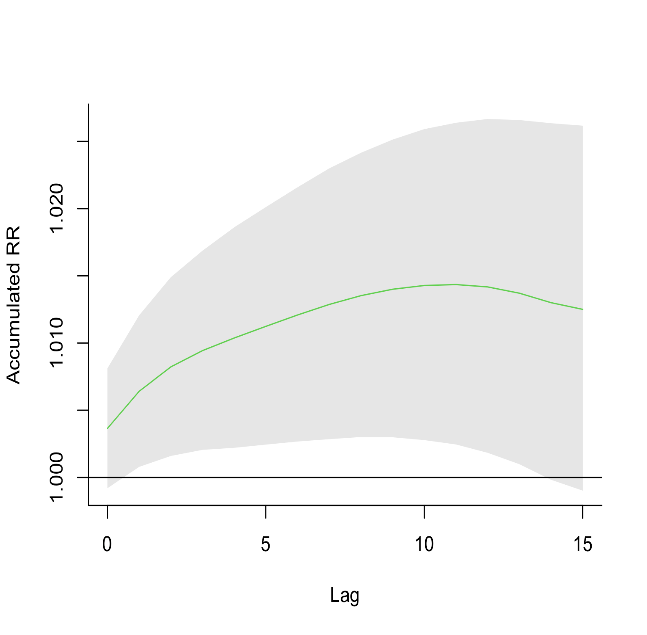** | **Norte de Santander**  **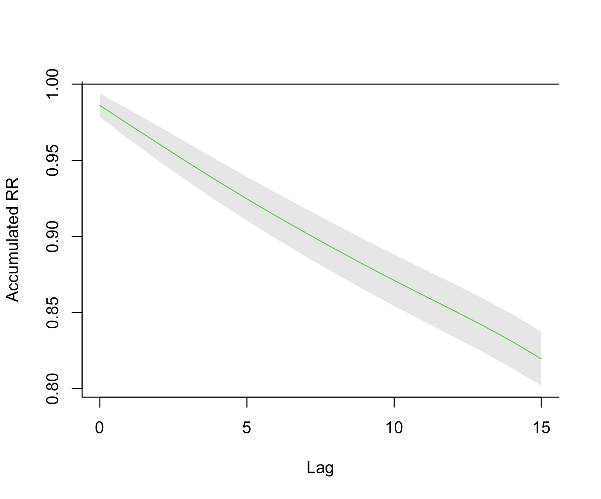** |
| **Boyacá**  **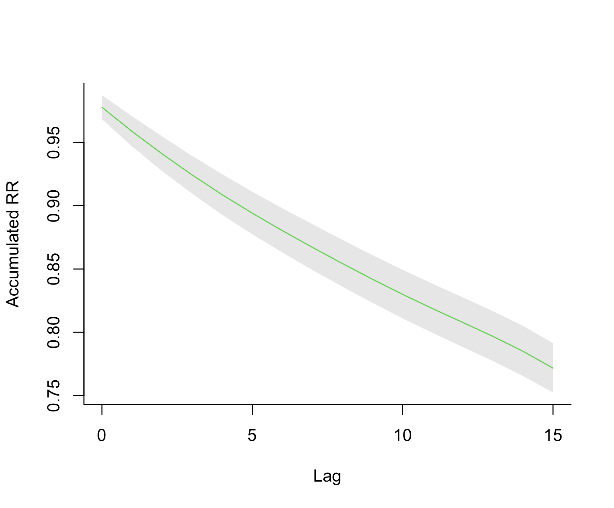** | **Bogotá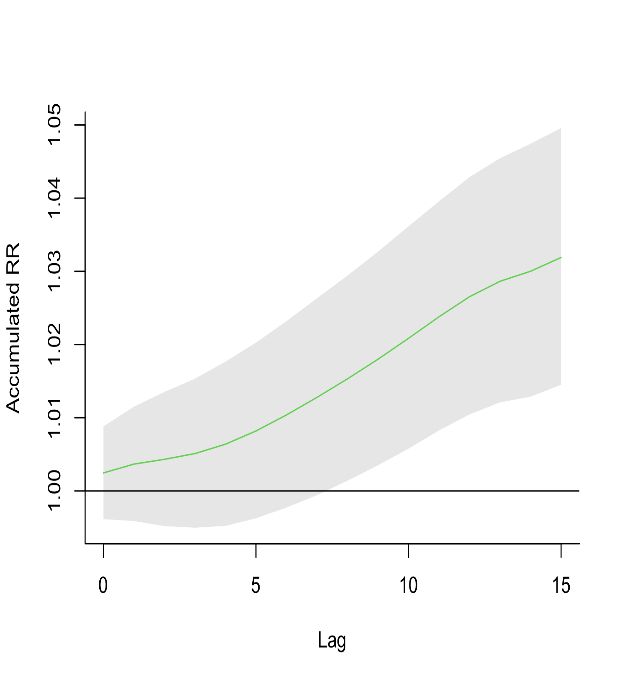** |
|  | **Caldas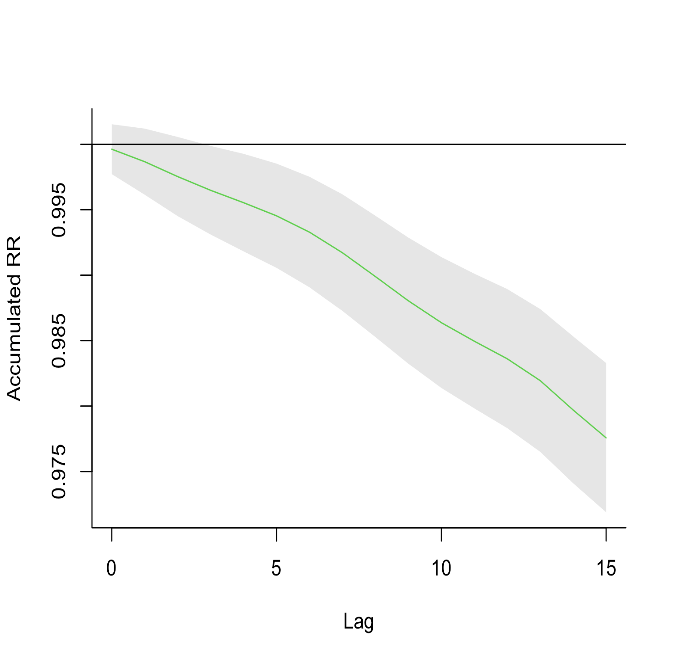** |
