# Supplementary material for: Exploring the association between precipitation and population cases of ocular toxoplasmosis in Colombia
Source: PLoS Negl Trop Dis. 2022 Oct 5;16(10):e0010742. doi: 10.1371/journal.pntd.0010742 (PMC9534415; doi:10.1371/journal.pntd.0010742)
Supplement: S2 Fig — (DOCX) [file pntd.0010742.s002.docx]

**S2 Figure:**  Summary of single-day lag-response curves for precipitation for subgroups at lag 0–14 used in each department of Colombia, 2015–2019.

| **Chocó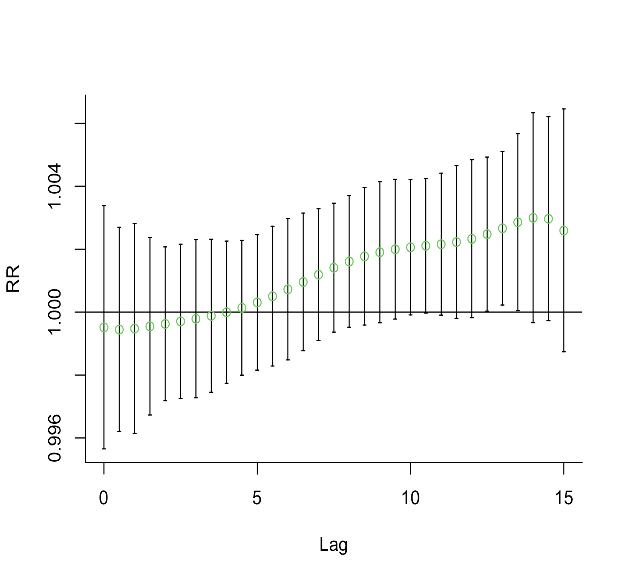** | **Tolima 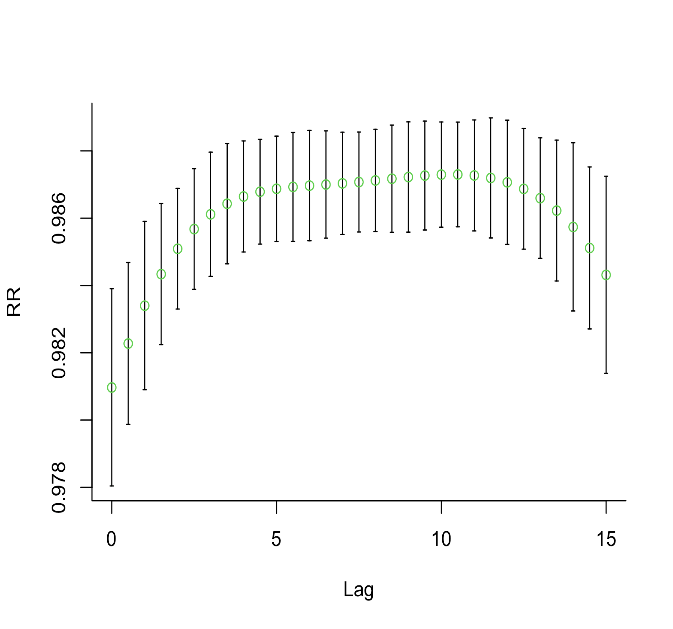** |
| --- | --- |
| **Antioquia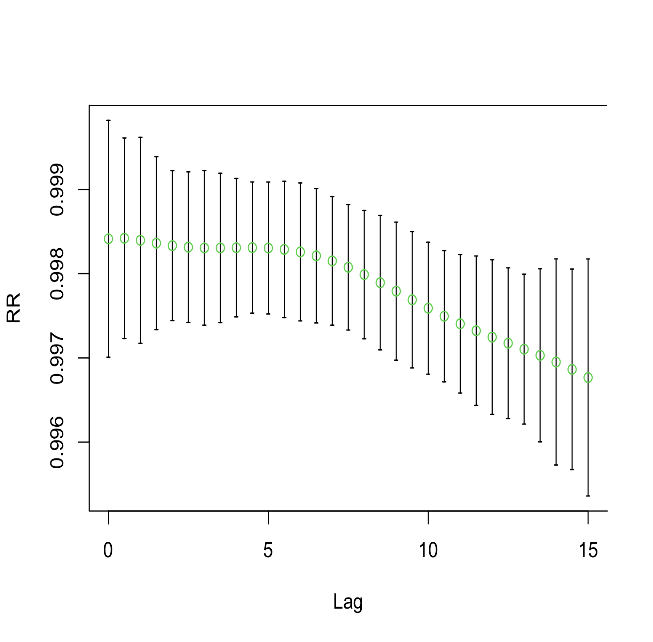** | **Cundinamarca**  **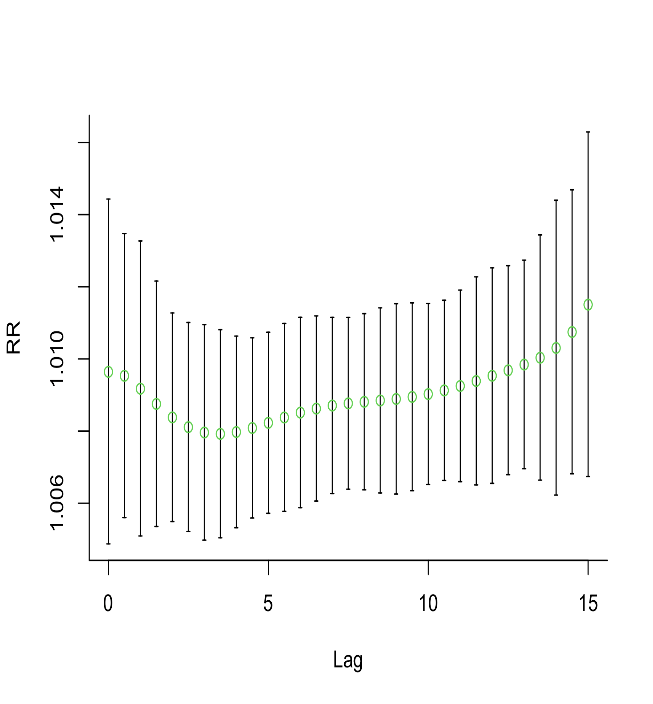** |
| **Guajira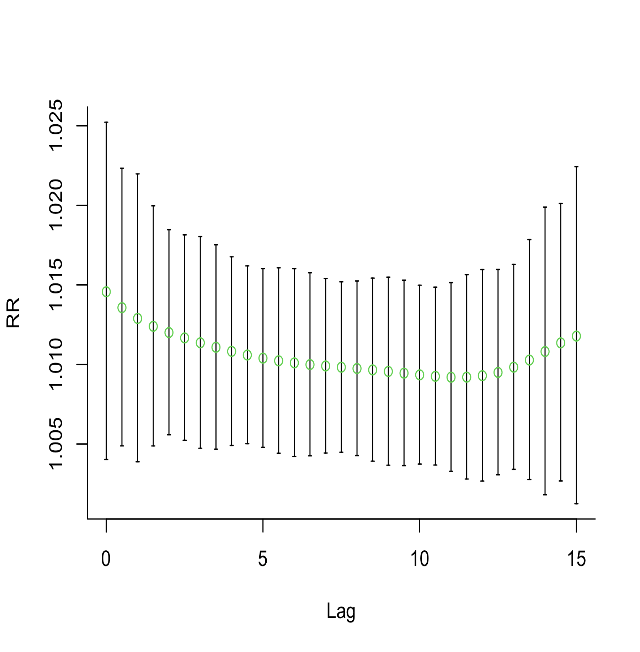** | **San Andrés**  **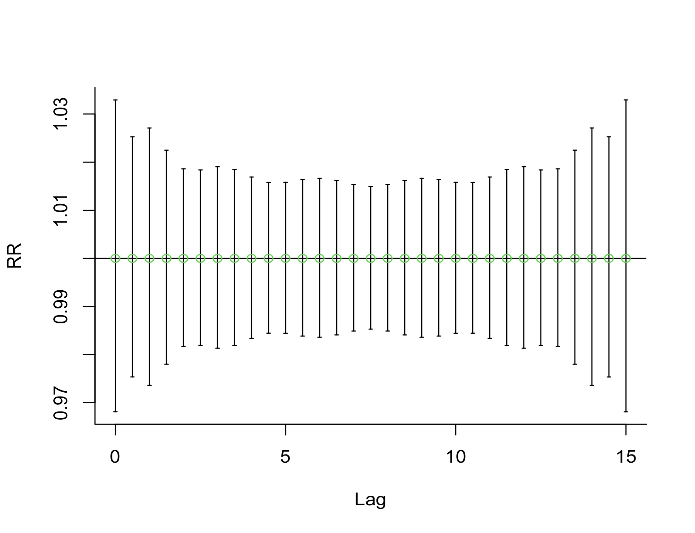** |
| **Boyacá 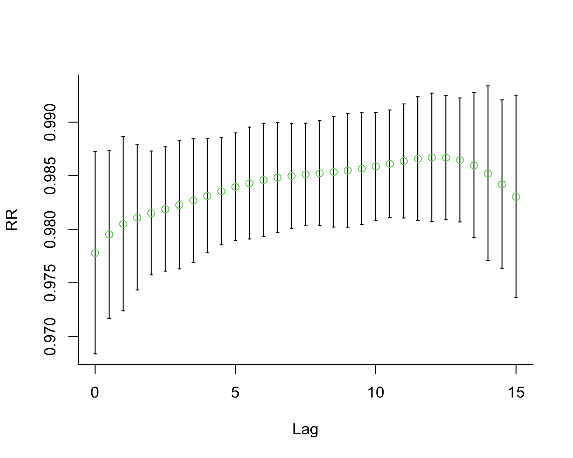** | **Caldas**  **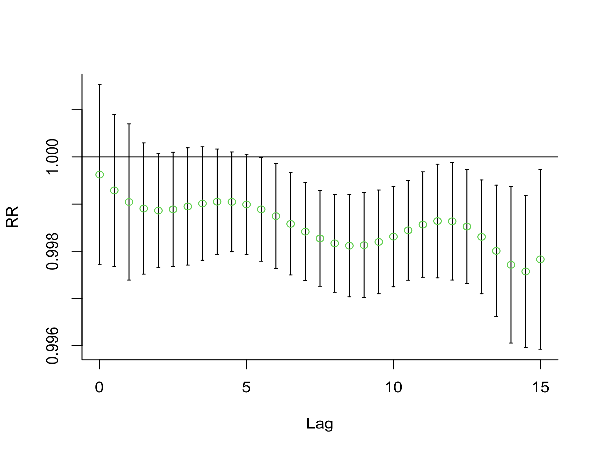** |
| **Cauca 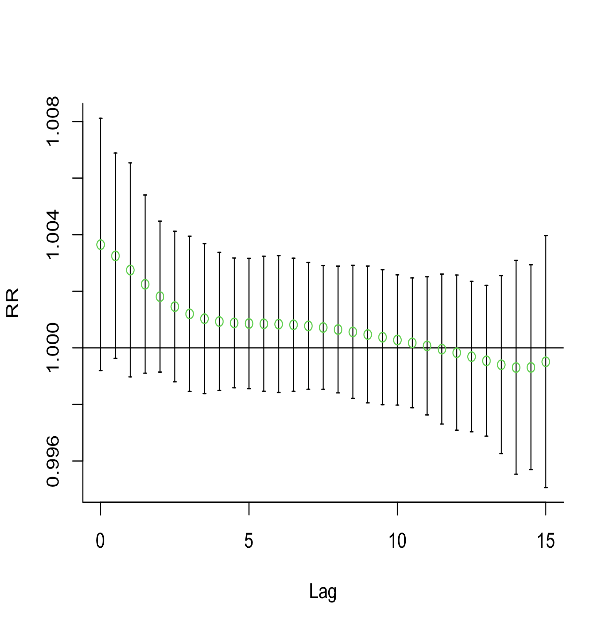** | **Bogotá**  **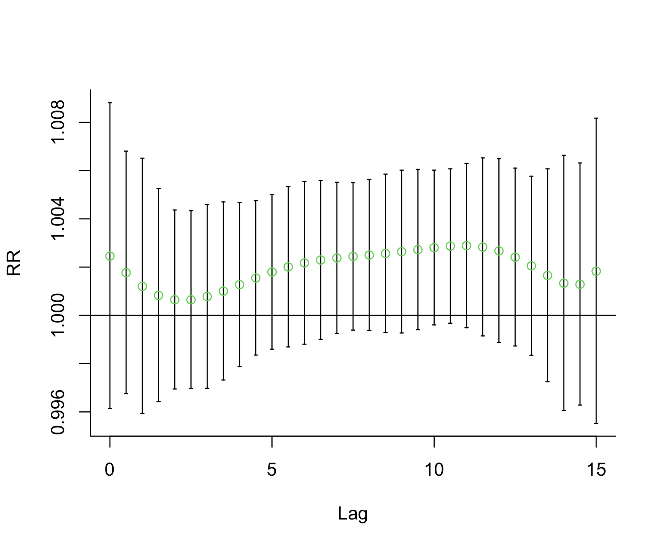** |
| **Córdoba**  **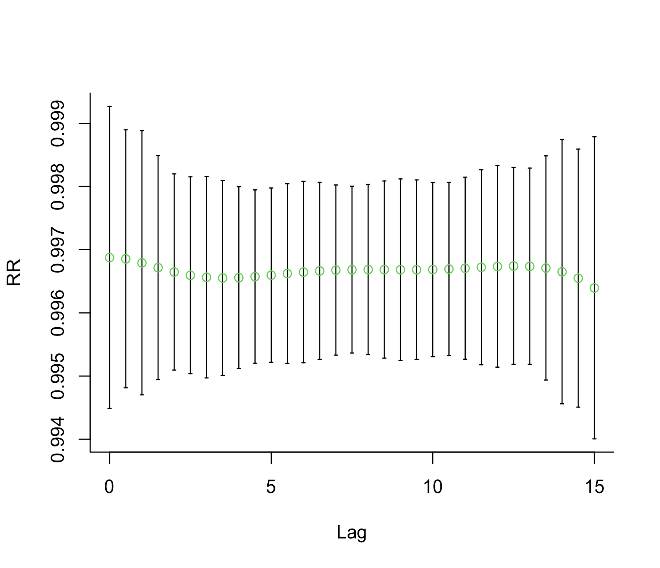** | **Bolívar 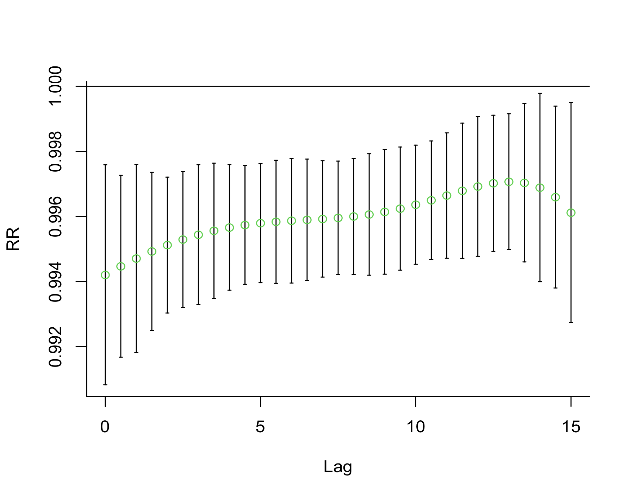** |
| **Sucre 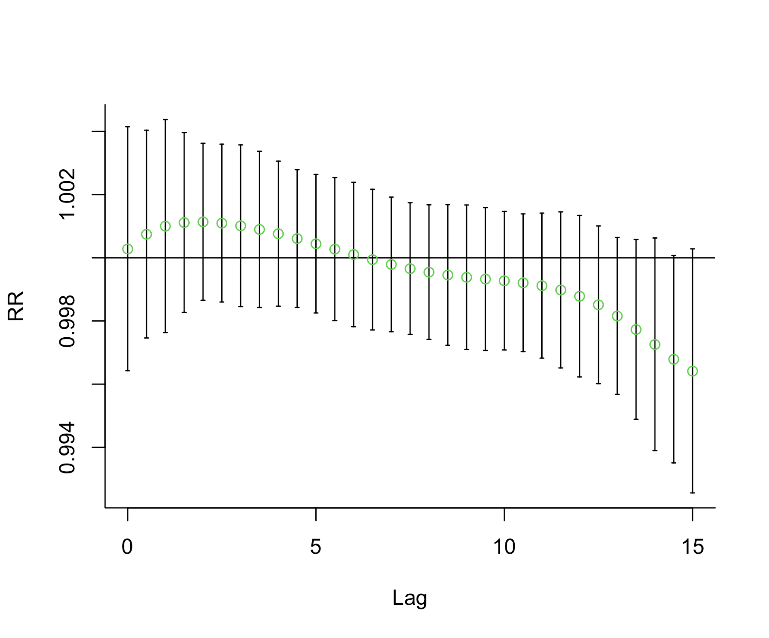** | **Magdalena 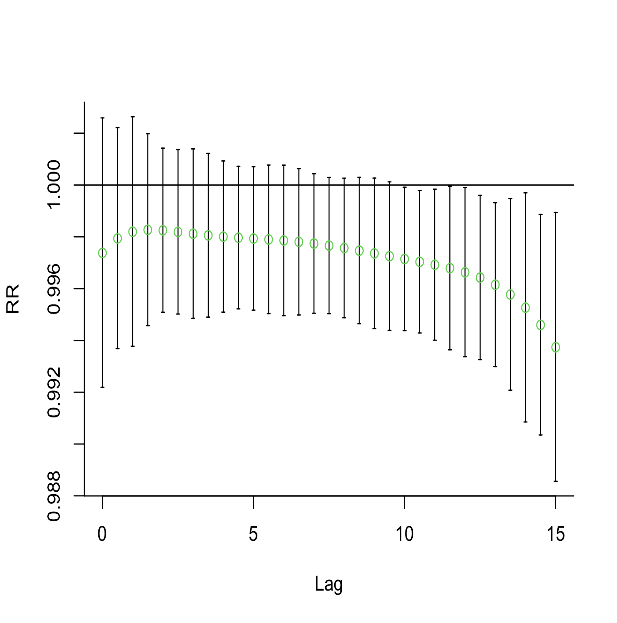** |
| **Atlántico 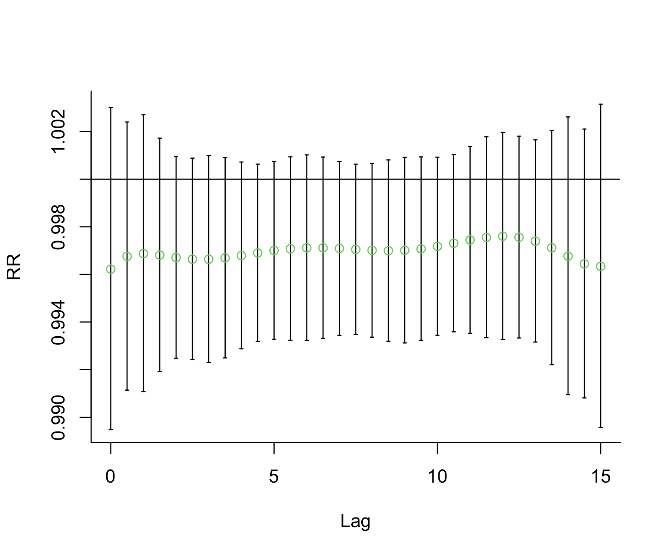** | **Cesar 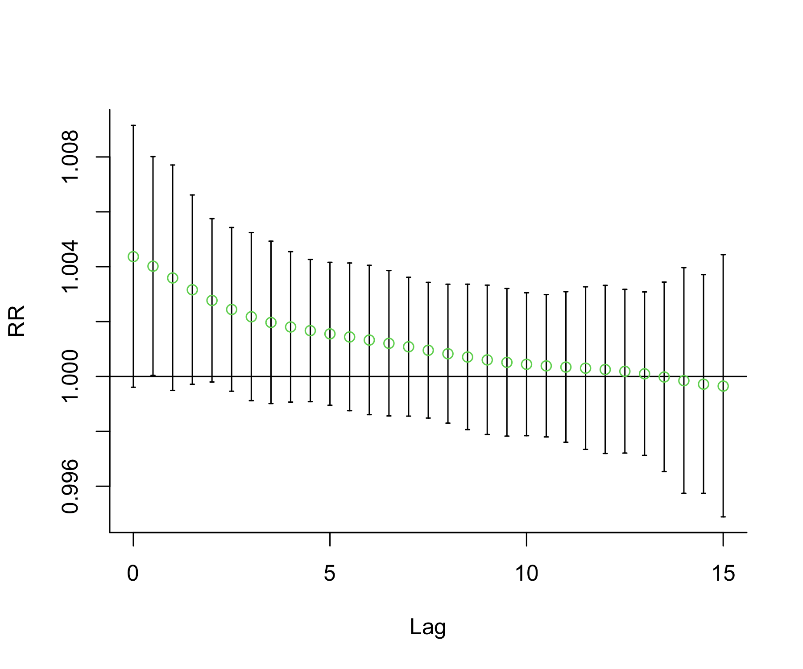** |
| **Huila**  **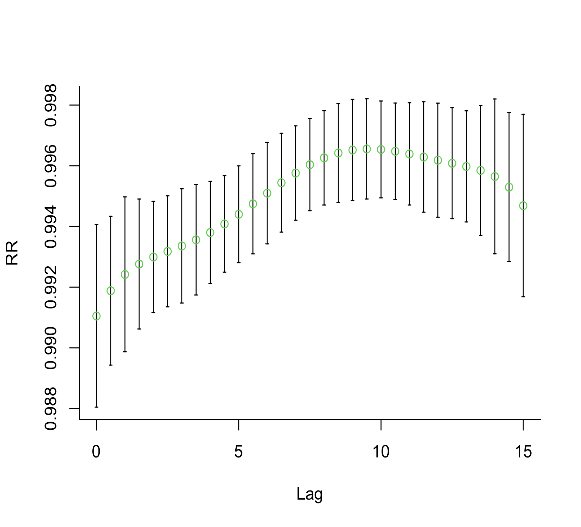** | **Norte de Santander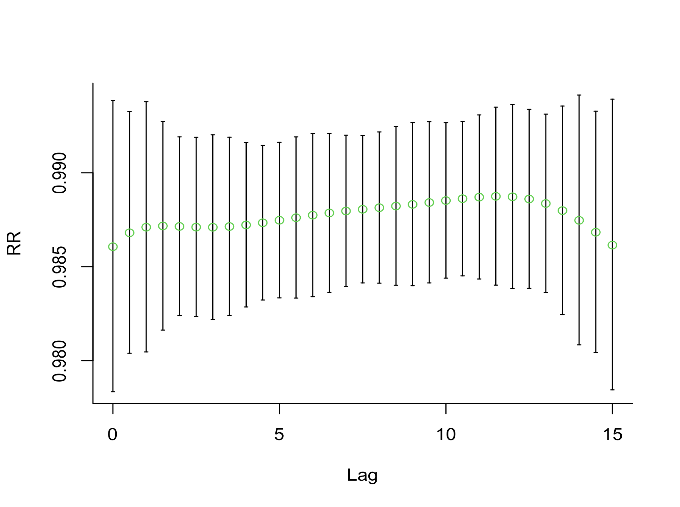** |
|  | **Santander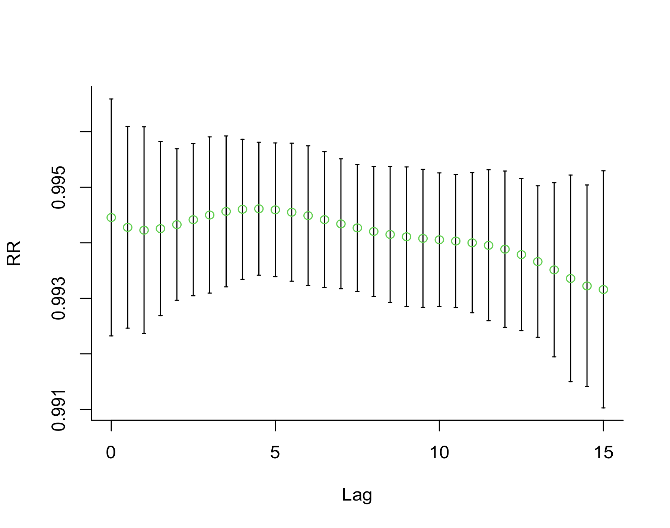** |
