# Supplementary material for: Exploring the association between precipitation and population cases of ocular toxoplasmosis in Colombia
Source: PLoS Negl Trop Dis. 2022 Oct 5;16(10):e0010742. doi: 10.1371/journal.pntd.0010742 (PMC9534415; doi:10.1371/journal.pntd.0010742)
Supplement: S1 Table — (DOCX) [file pntd.0010742.s003.docx]

**S1 Table:** Mean of Relative risk (RR) and 95% confidence intervals (95% CI) in each department of Colombia of ocular toxoplasmosis cases.

| Department | **lag 0- 6**  **Mean of relative risk RR (95% CI)** | **lag 6-10**  **Mean of relative risk RR (95% CI)** | **lag 10 – 12**  **Mean of relative risk RR (95% CI)** | **lag 13 – 15**  **Mean of relative risk RR (95% CI)** |
| --- | --- | --- | --- | --- |
| Choco | 0.9998(0.998-1) | 1(0.999-1) | 1.001(1-1) | 1.001(1-1) |
| Antioquia | 0.9992(0.999-1) | 0.9991(0.999-1) | 0.9988(0.998-0.999) | 0.9986(0.998-0.999) |
| Guajira | 1.007(1-1.01) | 1.005(1-1.01) | 1.005(1-1.01) | 1.005(1-1.01) |
| Tolima | 0.9905(0.989-0.992) | 0.9935(0.993-0.994) | 0.9936(0.993-0.994) | 0.9933(0.992-0.994) |
| Cundinamarca | 1.005(1-1.01) | 1.004(1-1.01) | 1.005(1-1.01) | 1.005(1-1.01) |
| Córdoba | 0.9984(0.997-1) | 0.9983(0.998-0.999) | 0.9983(0.998-0.999) | 0.9984(0.998-0.999) |
| Sucre | 1(0.998-1) | 1(0.999-1) | 0.9996(0.999-1) | 0.9991(0.998-1) |
| Atlántico | 0.9981(0.995-1) | 0.9986(0.997-1) | 0.9986(0.997-1) | 0.9987(0.997-1) |
| Bolívar | 0.9971(0.995-0.999) | 0.9979(0.997-0.999) | 0.9982(0.997-0.999) | 0.9985(0.997-1) |
| Magdalena | 0.9987(0.996-1) | 0.9989(0.997-1) | 0.9986(0.997-1) | 0.9981(0.996-1) |
| Cesar | 1.002(1-1) | 1.001(0.999-1) | 1(0.999-1) | 1(0.999-1) |
| Norte de Santander | 0.993(0.989-0.997) | 0.9939(0.992-0.996) | 0.9943(0.992-0.996) | 0.9942(0.992-0.997) |
| San Andrés | 1(0.984-1.02) | 1(0.992-1.01) | 1(0.992-1.01) | 1(0.991-1.01) |
| Huila | 0.9955(0.994-0.997) | 0.9975(0.997-0.998) | 0.9983(0.997-0.999) | 0.998(0.997-0.999) |
| Cauca | 1.002(1-1) | 1(0.999-1) | 1(0.999-1) | 0.9998(0.998-1) |
| Bogotá | 1.001(0.998-1) | 1.001(0.999-1) | 1.001(1-1) | 1.001(0.999-1) |
| Caldas | 0.9998(0.999-1) | 0.9994(0.999-1) | 0.9992(0.999-1) | 0.9992(0.999-1) |
| Boyacá | 0.9888(0.984-0.994) | 0.9923(0.99-0.995) | 0.9929(0.99-0.995) | 0.9932(0.99-0.996) |
| Santander | 0.9972(0.996-0.998) | 0.9972(0.997-0.998) | 0.997(0.996-0.998) | 0.9968(0.996-0.998) |
