# Supplementary material for: Exploring the association between precipitation and population cases of ocular toxoplasmosis in Colombia
Source: PLoS Negl Trop Dis. 2022 Oct 5;16(10):e0010742. doi: 10.1371/journal.pntd.0010742 (PMC9534415; doi:10.1371/journal.pntd.0010742)
Supplement: Attachment — Submitted filename: Answer for reviewers.pdf [file pntd.0010742.s004.pdf]

Bogotá, July 12, 2022

Dear Editor in Chief,

Thank you for the comments and corrections to the manuscript number PNTD-D-22-00336 entitled "*Exploring the association between precipitation and population cases of ocular toxoplasmosis in Colombia* ". We have attentively read the Editorial office and reviewers' comments and suggestions and amended them in the manuscript.

Editor and Reviewer comments:

Key Review Criteria Required for Acceptance?

As you describe the new analyses required for acceptance, please consider the following:

Methods
-Are the objectives of the study clearly articulated with a clear testable hypothesis stated?
-Is the study design appropriate to address the stated objectives?
-Is the population clearly described and appropriate for the hypothesis being tested?
-Is the sample size sufficient to ensure adequate power to address the hypothesis being tested?
-Were correct statistical analysis used to support conclusions?
-Are there concerns about ethical or regulatory requirements being met?

Reviewer #1: There is a clear objective for this study, but no state hypothesis from the authors. Additionally the authors need to make clear if there was any missing precipitation data and the potential impacts of missing precipitation data. the authors also need to account for population differences in the geographical regions of interest presented in this study.

Answer: Thank you for your valuable suggestions. We added information about the hypothesis on lines 122-123. The information about the impacts of missing precipitation data (exclusion of analysis) can be found on lines 190-194 and in the limitations section. To handle this limitation we performed a sub-analysis with the data available for 2019 (Lines 316-347). Also, the information about the population in the different geographical regions was added in the new Table 1.

Reviewer #2: (No Response)

Results

-Does the analysis presented match the analysis plan?
-Are the results clearly and completely presented?
-Are the figures (Tables, Images) of sufficient quality for clarity?

Reviewer #1: the results followed and analyses discussed in the methods section. Figure 1 is not clearly presented. Supplemental material 1 and 2 should be included as in-text tables for this manuscript.

Answer: Thank you for your suggestion, the changes were made in the results section.

Reviewer #2: (No Response)

Conclusions

-Are the conclusions supported by the data presented?
-Are the limitations of analysis clearly described?
-Do the authors discuss how these data can be helpful to advance our understanding of the topic under study?
-Is public health relevance addressed?

Reviewer #1: the authors conclusions are supported by the results. Additionally the authors do a nice job discussing the limitations of their work and data.

Answer: Thank you for your comment.

Reviewer #2: (No Response)

Editorial and Data Presentation Modifications?

Use this section for editorial suggestions as well as relatively minor modifications of existing data that would enhance clarity. If the only modifications needed are minor and/or editorial, you may wish to recommend "Minor Revision" or "Accept".

Reviewer #1: (No Response)

Reviewer #2: (No Response)

Summary and General Comments

Use this section to provide overall comments, discuss strengths/weaknesses of the study, novelty, significance, general execution and scholarship. You may also include additional comments for the author, including concerns about dual publication, research ethics, or publication ethics. If requesting major revision, please articulate the new experiments that are needed.

Reviewer #1: This study evaluated the relationship between precipitation and ocular toxoplasmosis incidence. I have some comments below on your manuscript:

a. Introduction: The authors should consider adding more information on the protozoan lifecycle and biology that allows it survive in conditions outside of its hosts. Additionally the authors should discuss routes of infection and what can influence infection.

Answer: Thank you for your kind suggestion, we have included more information on this topic in the introduction section (Lines 88-98).

b. Methods: the authors should note what months and years they gathered data for in regards to their outcome data (population data section) in a similar way it is mentioned in the precipitation data.

Answer: Thank you for your suggestion. We clarified this point in the methods section (Lines 166-170).

c. Methods: The authors state “825 files” were selected on line 148. Do these files represent data from 825 unique stations for the four years they were interested in researching? Or is it 825 unique files with data from all weather stations available?

Answer: Thank you for your comment. We have made a clearer description in lines 175-180.

d. Methods: the authors should state if there was missing data in regards to precipitation data, and if there was missing data how was in handled?

Answer: Thank you for bringing this to our attention. We could not obtain information from all the stations of the National Institute of Hydrology, Meteorology, and Environmental Studies (IDEAM), this was explained in lines 190-194 (statistical analysis section) and in lines 441-446 (limitations). However, to handle this limitation we performed a sub-analysis with the data available for 2019 (Lines 316-347).

e. Methods: the authors should make remarks on how they accounted for population size differences between geographic regions that could account for some differences they see in counts of OT per region.
Answer: Thanks for your suggestion. We include this in lines 135-140 and Table 1.

f. Results: Supplementary Material 1 and 2 should be included as in-text tables as they describe the authors data used for their results
Answer: Thank you for your recommendation. We modified Table 1 with the information from previous supplementary materials 1 and 2.

g. Results: In line 209, the authors describe different departments. I believe departments is referring to geographical regions of Columbia. The authors should make this more clear throughout the manuscript and provide greater discussion in their methods that these are analyses are performed for each geographic region.
Answer: Thanks for the suggestion, in the methods section we added information about the term “departments” and regarding the limitation of the analyses performed for some geographic regions. (Lines 136-138, 183-194, 219-230)

h. Results: the authors should give a greater description of what a characteristic pattern is for line 217.
Answer: Thank you for your suggestion, it was done on lines 255-268.

i. Results: The legend in figure 4B would indicate that precipitation is associated with a decrease in risk since RR < 1. Is the legend presented in this figure accurate?

Answer: Thank you for your observation. Figure 4 and its legend were modified to be clearer.

j. Results: in line 273-275 the authors should state there is an association between precipitation and increased OT rather than the causal language currently used. Direct causality is difficult to prove and assess in a population study.
Answer: Thank you for your observation. We rephrased the paragraph (Lines 318-325).

k. Results: For data presented in supplementary Material 2, is this average of daily averages, or the average of hourly precipitation recorded from weather stations? Authors should make this clearer.
Answer: Thank you for your question. Indeed, we refer to the average of daily averages. We converted Supplementary materials 1 and 2 in Table 1 and explained how the “daily mean” was calculated (Line 177-180).

l. Results: Figure 1 is difficult to read the graphs. Either splitting the map into northern and southern compartment to make a two part figure would be helpful. Or placing an asterisk (or some denoting

feature) on the 7 characteristic patterns would provide improved readability of these graphs. Or decreasing the size of the map and increasing the graphs.

Answer: Thanks for your suggestions, figure 1 was modified. It was divided, increasing the size of the graphs and adding identification numbers to seven departments of interest.

m. Results / Discussion: Authors should discuss why in some regions there is large variability in RR (Cauca, Sucre, San Andres, etc.) where in others there is very little RR variability

Answer: Thank you for your suggestion, it was done on lines 406-420.

Reviewer #2: In this manuscript, the authors describe the association between precipitation and occurrence of ocular toxoplasmosis (OT) cases in different location in Colombia. OT is a serious clinical problem in South America, and studies to understand the interaction between condition and its environment are more than welcome to enable efficient public health measures on a local level. As previous studies (cited in the manuscript) showed, precipitation is one of the important factors. This kind of study is well adapted for publication in PLOS Neglected Tropical Diseases. The senior author has a solid publication list in OT clinical and basic science. The authors collected a huge data set to explain different ways of how precipitation affects OT cases, more than enough to justify an article. Unfortunately, the manuscript is written in a language and style apparently destined to meteorology experts, including a lot of formulas, techniques and interpretations specific to this area. In my opinion, an article in PLOS NTD should be accessible to readers trained in infectiology and tropical health science. Being such a reader, I considerably struggled to understand the methods, results and conclusions of the study. So, to be able to evaluate the manuscript correctly, I suggest the following points:

1 Clearly explain the methods used, and the terms employed for interpretation (lag time…). For example, the authors state that the filter … was applied for each year (l. 140), but the term 'year' never appeared in the results. In l. 154, it should be explained what exactly means 'case period' and 'nearby referent periods'. The two following sentences are, as generally all this statistical analysis (= methods sensu stricto), very difficult to understand for the average infection/tropical health reader. The second paragraph of this section seems to be important, but should be clearly explained. This does, of course, not exclude some technical formulas, when well explained.

Answer: Thank you for your suggestions. We made changes throughout the manuscript, including methods and statistical analysis section. For example, the term "case period" was adjusted to be clearer: "In this study, we compared the exposure (daily average precipitation) in the period when events (cases of OT) occurred". In the same way, the term year does not appear in the results because we use the annual data to interpolate the daily cases and thus be able to simulate an observation window of 14 days, with which we can identify through the statistical model what happens with the RR after an exposure. Also, we rephrase some segments of the statistical analysis section" (Lines 219-228) to do the manuscript more understandable for infection/tropical health readers. Finally, we tried to reduce the complexity of the manuscript, maintaining the necessary information so that the procedures carried out were understandable.

Also, we want to explain in more detail the distributed lag non-linear models (DLNM), which is a methodology for simultaneously describing nonlinear and lag dependencies:

The DLNM modeling class is used to describe associations where the dependency between an exposure and an outcome is delayed in time, that is the lag. This lag dimension represents a new space over which the association is defined, by describing a lag-response relationship. So, you have two spaces: the exposure-response relationship and the lag-response relationship. Statistical development of DLNM is based on the application of basic functions to parameterize the exposure history, that is, the set of delayed exposures. In this paper, it's defined through a stratified design, that is, we do not know exactly on what day a person was exposed or became ill, but we can assume a series of scenarios through stratified sampling where this could occur. We used the calendar month as the time stratum, to control the effects of long-term trends, seasonality, and day of the week.

Then, two sets of basic functions are independently chosen to model the exposure and delayed response relationships. These are then combined through a special tensor product (a tensor describes a multilinear relationship between sets of algebraic objects) defined by two-dimensional cross-basis functions (one basis is for the lag and another is for the predictor). The choice of the two sets of functions determines the shape of the relationship in each dimension. Then, in simple words, you can use this product as a design matrix of a regression model.

The estimation is done using standard regression models and R functions, simply by including the matrix that stores the cross-basis variables in a model formula. The result can be interpreted by creating a grid of predictions for each lag.

2 The results are also difficult to decipher. The main findings should be explained in clear statements for each figure. The legend to Fig.3 is very difficult to understand, to give just one example.
Answer: Thank you for your suggestion, we changed the legends in the figures.

3 The text treating Fig4 and Table 1 (l.270-277) is quite difficult to follow, for different reasons: The methods employed are not clearly explained to the average reader (cf. point 1), the language of some sentences lack themselves clarity, e.g. l.272-273, and finally, the principal findings of the Table and Fig. are not clearly resumed in the text.
Answer: Thank you for your suggestion, all these comments were addressed throughout the manuscript. Additionally, we have sent the manuscript to English proofreading to Enago. We hope the fluency had improved.

4 The discussion also lacks clear interpretation of data, stating basically that rainfall can both be risky and protective, for unknown 'population behavior' (l. 311f) reasons. Apart from the wind, at the end of the discussion, none is really detailed, and maybe taken as example for specific districts.

Answer: Thank you for your suggestion, data interpretation was added (Lines 405-420).

In summary, I think that the manuscript contains valuable information, but should be explained in a more commonly understandable fashion and language.

Answer: Thanks for your comment. We try to explain in a more commonly understandable fashion and language. Additionally, we have sent the manuscript for English proofreading by Enago (UNIROL-158 for language and grammar), the letter is attached to this submission.

I have just some more general points:

5 Most of the cited articles relate high precipitation levels and toxoplasmosis outbreaks to failures of the filtration system in public water supply. Here, it should be at least discussed if more rain acts through longer oocyst survival or more oocysts leaking into the public water supply.
Answer: Thank you for your suggestion, this information was added in the discussion section (lines 369-376).

6 I admit that I did not entirely understand the meaning of the term 'delay' throughout the manuscript, but if this means that OT cases were observed with a delay of a few days after rainfall episodes, this has to be explained. Ocular affection usually takes time to develop following infection. Moreover, can new detected OT cases serve as measure for new general infections?

Answer: In statistics, the term "delay" is not used much, actually "lag" is used more often. We refer to "lag" or "delay" to the fact that the infection does not appear on the same day of exposure, instead, we assume that it may take at least 15 days to manifest. This was explained in the methods section.

In fact, in the reported OT outbreaks related to water contamination, the peaks of OT may start after 12 to 13 days of increased rainfall (Bowie, W. R., King, A. S., Werker, D. H., Isaac-Renton, J. L., Bell, A., Eng, S. B., & Marion, S. A. (1997). Outbreak of toxoplasmosis associated with municipal drinking water. The Lancet, 350(9072), 173–177. doi:10.1016/s0140-6736(96)11105-3).

7 Do the data allow to confirm the results in Ref 9 (Gomez-Marin et al.) of a correlation between rainfall and T. gondii infection rates in Colombian districts?

Answer: Thank you for your question. Unfortunately, with the data provided by the IDEAM, it was not possible to include Caquetá and Quindío in the dynamic analysis with the distributed lag non-linear model (DLNM). Therefore, to address this question we collected the average rainfall data for these departments from the page http://www.ideam.gov.co, just for the year 2019 (Data available). Then, a Pearson correlation test was performed for the available data, which showed that there is a correlation between precipitation and reported cases of toxoplasmosis with a value of 0.84 (*P* < 0.05), which confirms the hypothesis of the results in Ref 9 (Gomez-Marin et al.). A section with this analysis was added to the manuscript.

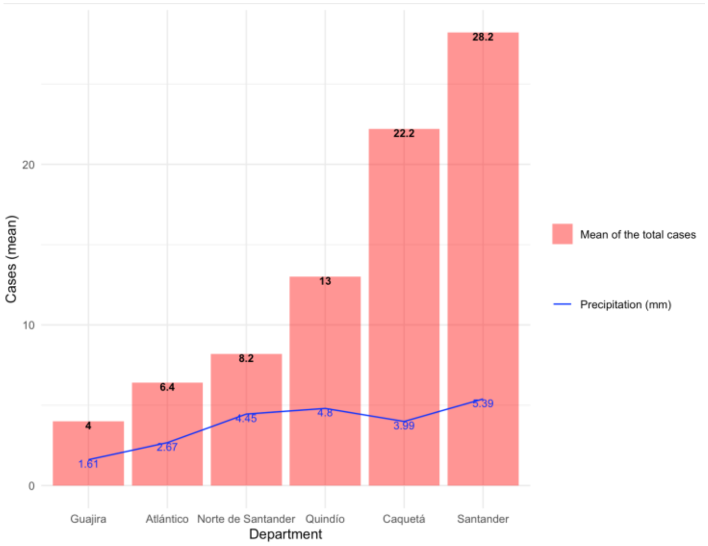

Figure: Total cases of toxoplasmosis and precipitation level by department.

Additionally, with the data obtained for 2019, we estimated a positive β coefficient (0.03) with P< 0.05 between precipitation and OT risk overall, which suggests that precipitation significantly influences the response variable (risk of OT) in all the evaluated departments.

Sincerely.

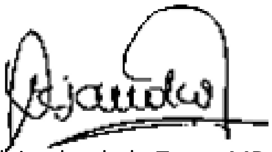

Alejandra de-la-Torre, MD, PhD

Corresponding author
